# Effects of angiotensin receptor-neprilysin inhibitor on ketone body metabolism in pre-heart failure/heart failure patients

**DOI:** 10.1038/s41598-024-67524-6

**Published:** 2024-07-17

**Authors:** Yusuke Kashiwagi, Tomohisa Nagoshi, Yoshiro Tanaka, Yuhei Oi, Haruka Kimura, Kazuo Ogawa, Makoto Kawai, Michihiro Yoshimura

**Affiliations:** Division of Cardiology, Department of Internal Medicine, The Jikei University School of Medicine, 3-25-8, Nishi-shimbashi, Minato-ku, Tokyo, 105-8461 Japan

**Keywords:** Angiotensin receptor-neprilysin inhibitor (ARNI), Ketone body, Heart failure, Cardiovascular biology, Heart failure

## Abstract

Recently, a mild elevation of the blood ketone levels was found to exert multifaceted cardioprotective effects. To investigate the effect of angiotensin receptor neprilysin inhibitors (ARNIs) on the blood ketone body levels, 46 stable pre-heart failure (HF)/HF patients were studied, including 23 who switched from angiotensin-converting enzyme (ACE) inhibitors or angiotensin receptor blockers (ARBs) to ARNIs (ARNI group) and 23 who continued treatment with ACE inhibitors or ARBs (control group). At baseline, there were no significant differences in the total ketone body (TKB) levels between the two groups. Three months later, the TKB levels in the ARNI group were higher than the baseline values (baseline to 3 months: 71 [51, 122] to 92 [61, 270] μmol/L, *P* < 0.01). In the control group, no significant change was observed between the baseline and 3 months later. A multiple regression analysis demonstrated that the initiation of ARNI and an increase in the blood non-esterified fatty acid (NEFA) levels at 3 months increased the percentage changes in the TKB levels from baseline to 3 months (%ΔTKB level) (initiation of ARNI: *P* = 0.017, NEFA level at 3 months: *P* < 0.001). These results indicate that ARNI administration induces a mild elevation of the blood TKB levels in pre-HF/HF patients.

## Introduction

In recent years, several studies have focused on the cardioprotective effects of ketone bodies^1,2^. Sodium glucose cotransporter 2 (SGLT2) inhibitors are now indispensable medications for heart failure, and the elevation of ketone body levels induced by these medicines is thought to contribute to their cardioprotective effects^3^.

Although fatty acids and glucose are mainly utilized as cardiac energy substrates in normal adult hearts, ketone bodies are an important preferential substrate under pathological conditions, such as heart failure^4–6^. In diabetic patients, the cardiac ketone body uptake increases as an energy source partially replacing glucose^7^. In addition to being an efficient fuel substrate in heart failure, ketone bodies are involved in multifaceted cardioprotective effects. Cardiac overexpression of beta-hydroxybutyrate dehydrogenase 1 (BDH1), which promotes the oxidation of ketone bodies, promotes increased ketone body utilization, decreases oxidative stress, and protects against heart failure^8^. Ketone bodies (β-hydroxybutyrate) inhibit NOD-like receptor protein 3 (NLPR3) inflammasome formation and antagonize pro-inflammatory cytokine-triggered mitochondrial dysfunction and fibrosis^9^. In addition, ketogenesis protects the energy-producing capacity of mitochondria by preventing hyperacetylation of mitochondrial proteins^10^. Although excessively elevated total ketone body (TKB) levels may cause ketoacidosis, which may have fatal consequences, intentionally induced mild ketonemia is likely to exert cardioprotective effects.

Angiotensin receptor-neprilysin inhibitor (ARNI) reduced cardiovascular deaths and all-cause mortality compared with angiotensin-converting enzyme (ACE) inhibitors in patients with chronic heart failure and a reduced ejection fraction (HFrEF)^11^. The main reason for this cardioprotective effect of ARNI on HFrEF is considered to be the improvement in the cardiac function via reverse cardiac remodeling^12,13^. We and others have demonstrated that natriuretic peptides (NPs), which are increased by neprilysin inhibition, can play a crucial role in energy metabolism^14–17^, so ARNI may also exert a significant effect on energy metabolism. Previous studies have reported that treatment with ARNI resulted in improved glucose metabolism^18,19^. We also found that ARNI administration improved insulin resistance in patients with stable chronic heart failure^20^. However, the mechanism by which ARNI affects ketone body metabolism is not yet fully understood.

In the present study, we investigated the effect of ARNI on blood TKB levels in pre-heart failure/heart failure patients and the possible mechanisms underlying the relationship between ARNI and ketone body metabolism.

## Results

### A comparison of the clinical characteristics of the two groups at baseline

Table 1 and Supplementary Table S1-S3 show the clinical characteristics of the patients at baseline in this study. The left ventricular ejection fraction (LVEF) of the ARNI group was lower than that of the control group (LVEF, 51.9 ± 2.53 vs. 60.7 ± 1.58%, *P* = 0.005). The body mass index (BMI) and fasting blood insulin level in the ARNI group were significantly higher than in the control group (BMI, 24.7 [23.5, 27.4] vs. 23.7 [21.0, 25.3] kg/m^2^, *P* = 0.027; fasting blood insulin, 9.6 [6.3, 12.7] vs. 5.6 [3.3, 8.0] μU/mL, *P* = 0.0002). There were no significant differences in TKB levels between the two groups. Furthermore, there were no significant differences in the administration rates of medications for heart failure or antidiabetic agents between the two groups.

**Table 1:** Clinical characteristics of the patients at baseline.

Characteristics	Number (%), mean ± SE or median (25th, 75th percentile)		P
	ARNI group (n = 23)	Control group (n = 23)	
Male (n, %)	19 (82.6)	17 (73.9)	NS
Age (years)	63.2 ± 2.47	69.0 ± 2.22	NS
BMI (kg/m^2^)	24.7 (23.5, 27.4)	23.7 (21.0, 25.3)	0.027
SBP (mmHg)	129 ± 3.48	129 ± 2.87	NS
DBP (mmHg)	77.7 ± 2.28	79.4 ± 2.64	NS
Heart rate (beats per minute)	75.8 ± 2.54	76.4 ± 3.22	NS
LVEF (%)	51.9 ± 2.53	60.7 ± 1.58	0.005
FBS (mg/dL)	110 (100, 141)	102 (93, 122)	NS
Fasting blood insulin (µU/mL)	9.6 (6.3, 12.7)	5.6 (3.3, 8.0)	0.0002
HbA1c (%)	6.3 (5.7, 6.6)	6.1 (5.8, 6.4)	NS
TG (mg/dL)	126 (100, 176)	111 (73, 137)	NS
HDL (mg/dL)	61.3 ± 3.53	59.4 ± 2.60	NS
LDL (mg/dL)	102 ± 6.79	96.2 ± 6.26	NS
AST (U/L)	25 (22, 27)	26 (21, 28)	NS
ALT (U/L)	27 (22, 33)	22 (17, 25)	0.043
LDH (U/L)	175 (167, 198)	183 (170, 195)	NS
ALP (U/L)	72.8 ± 5.66	76.4 ± 5.24	NS
γ-GTP (U/L)	50 (41, 62)	34 (18, 57)	0.027
TB (mg/dL)	0.7 (0.5, 0.8)	0.6 (0.5, 0.9)	NS
Albumin (g/dL)	4.42 ± 0.07	4.27 ± 0.09	NS
eGFR (mL/min/1.73 m^2^)	64.9 ± 3.75	58.8 ± 3.71	NS
UA (mg/dL)	6.44 ± 0.27	6.27 ± 0.30	NS
BNP (pg/mL)	17.1 (8.7, 69.9)	46 (18.7, 109)	NS
NEFA (μEq/L)	545 ± 48.6	542 ± 47.8	NS
TKB (μmol/L)	71 (51, 122)	59 (45, 84)	NS
Underlying main cardiovascular diseases			
Ischemic heart disease (n, %)	12 (52.2)	12 (52.2)	NS
Cardiomyopathy (n, %)	8 (34.8)	2 (8.7)	0.032
Arrhythmia (n, %)	2 (8.7)	8 (34.8)	0.032
Valvular disease (n, %)	0 (0)	1 (4.3)	NS
Congenital heart disease (n, %)	1 (4.3)	0 (0)	NS
Comorbidities			
Diabetes mellitus (n, %)	7 (30.4)	3 (13.0)	NS
Hypertension (n, %)	19 (82.6)	17 (73.9)	NS
Dyslipidemia (n, %)	20 (87.0)	20 (87.0)	NS
Renal dysfunction (n, %)	8 (34.8)	12 (52.2)	NS
Atrial fibrillation (n, %)	1 (4.3)	5 (21.7)	NS
Medications			
Beta-blockers (n, %)	17 (73.9)	20 (87.0)	NS
MRAs (n, %)	11 (47.8)	5 (21.7)	NS
Diuretics (n, %)	10 (43.5)	11 (47.8)	NS
SGLT2 inhibitors (n, %)	2 (8.70)	2 (8.70)	NS
Lipid-lowering drugs (n, %)	19 (82.6)	18 (78.3)	NS
Antidiabetic agents* (n, %)	5 (21.7)	2 (8.70)	NS

SE, standard error; ARNI, angiotensin receptor-neprilysin inhibitor; BMI, body mass index; SBP, systolic blood pressure; DBP, diastolic blood pressure; LVEF, left ventricular ejection fraction; FBS, fasting blood sugar; Hb, hemoglobin; TG, triglyceride; HDL, high-density lipoprotein cholesterol; LDL, low-density lipoprotein cholesterol; AST, aspartate aminotransferase; ALT, alanine aminotransferase; LDH, lactate dehydrogenase; ALP, alkaline phosphatase; γ-GTP, γ-glutamyltranspeptidase; TB, total bilirubin; eGFR, estimated glomerular filtration rate; UA, uric acid; BNP, B-type natriuretic peptide; NEFA, non-esterified fatty acid; TKB, total ketone body; MRA, mineralocorticoid receptor antagonist; SGLT, sodium-glucose cotransporter.

*Antidiabetic agents include SGLT2 inhibitors.

### Changes in clinical factors from baseline to three months

**Figure 1:**
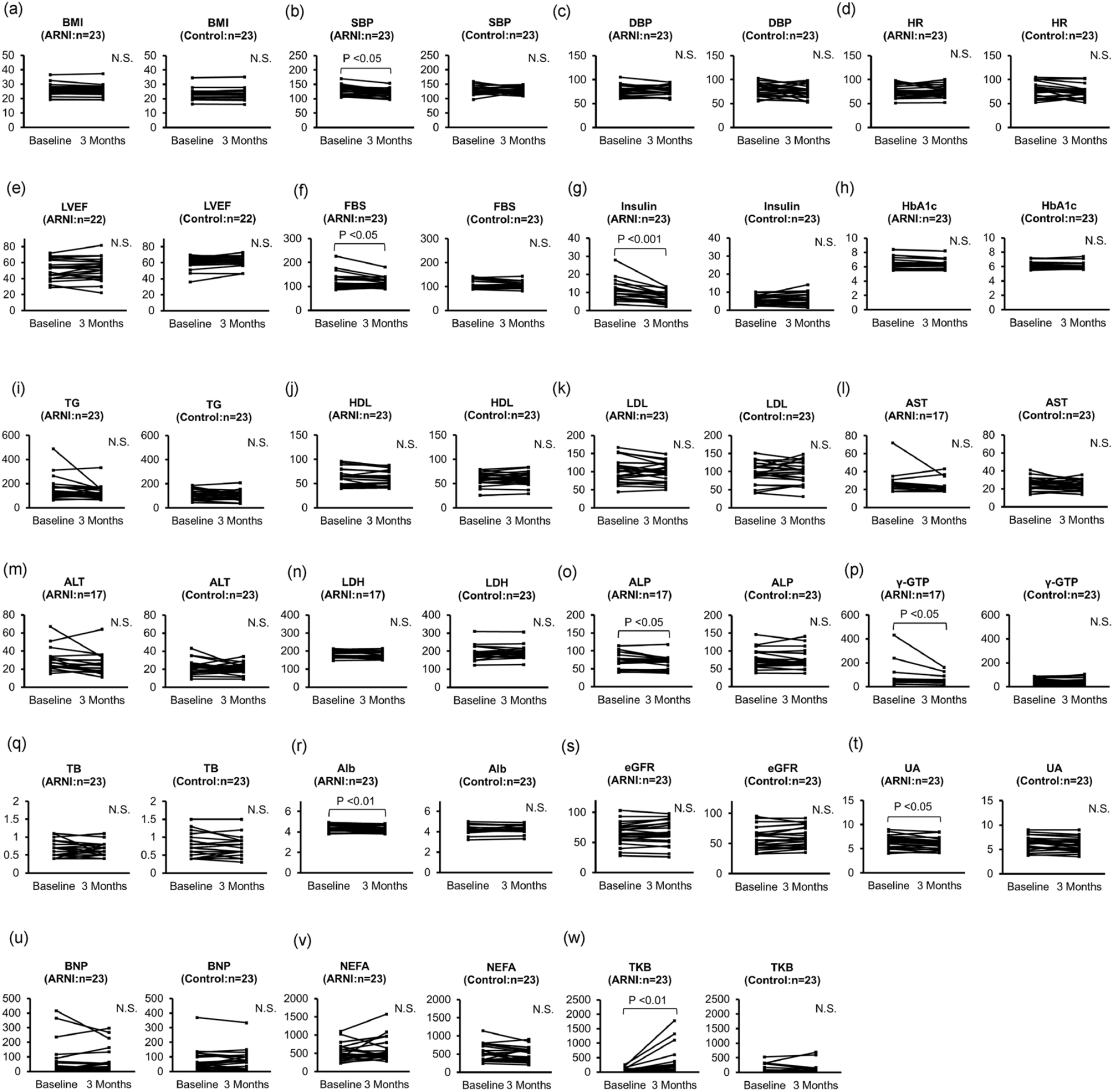
Changes in clinical factors from baseline to 3 months later the initiation of ARNI in patients with pre-heart failure/heart failure. (**a**) BMI, (**b**) SBP, (**c**) DBP, (**d**) HR, (**e**) LVEF, (**f**) FBS, (**g**) Insulin, (**h**) HbA1c, (**i**) TG, (**j**) HDL, (**k**) LDL, (**l**) AST, (**m**) ALT, (**n**) LDH, (**o**) ALP, (**p**) γ-GTP, (**q**) TB, (**r**) Alb, (**s**) eGFR, (**t**) UA, (**u**) BNP, (**v**) NEFA, (**w**) TKB. N.S., not significant; ARNI, angiotensin receptor-neprilysin inhibitor; BMI, body mass index; SBP, systolic blood pressure; DBP, diastolic blood pressure; HR, heart rate; LVEF, left ventricular ejection fraction; FBS, fasting blood sugar; Hb, hemoglobin; TG, triglyceride; HDL, high-density lipoprotein cholesterol; LDL, low-density lipoprotein cholesterol; AST, aspartate aminotransferase; ALT, alanine aminotransferase; LDH, lactate dehydrogenase; ALP, alkaline phosphatase; γ-GTP, γ-glutamyltranspeptidase; TB, total bilirubin; Alb, albumin; eGFR, estimated glomerular filtration rate; UA, uric acid; BNP, B-type natriuretic peptide; NEFA, non-esterified fatty acid; TKB, total ketone body.

**Figure 2:**
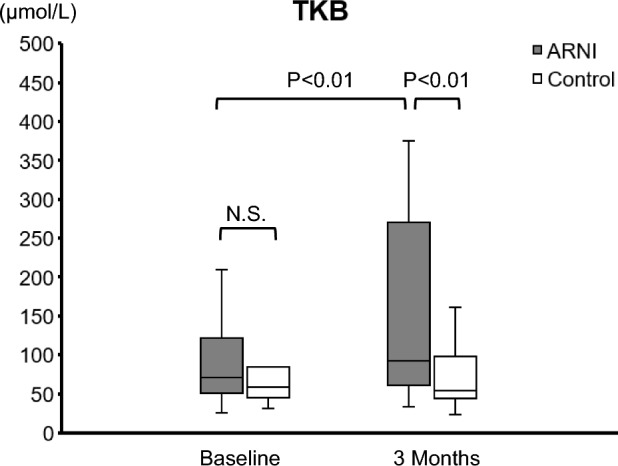
The comparison of the TKB level between the ARNI group and control group at baseline and three months. TKB, total ketone body; ARNI, angiotensin receptor-neprilysin inhibitor.

Figure 1 shows that, at three months, the systolic blood pressure, fasting blood sugar, fasting blood insulin, and uric acid (UA) values had decreased, while the TKB levels had increased in comparison to the baseline levels in the ARNI group (baseline to 3 months: systolic blood pressure, 129 ± 3.48 to 123 ± 2.70 mmHg, *P* = 0.036; blood sugar, 110 [100, 141] to 102 [99, 128] mg/dL, *P* = 0.013; insulin, 9.6 [6.3, 12.7] to 7.6 [4.7, 9.3] μU/mL, *P* < 0.001; UA, 6.44 ± 0.27 to 6.07 ± 0.27 mg/dL, *P* = 0.017 and TKB level, 71 [51, 122] to 92 [61, 270] μmol/L, *P* < 0.01). In the control group, there were no significant changes between baseline and 3 months after treatment for any of the clinical factors. In addition, at baseline, there was no marked difference in TKB levels between the two groups; however, after three months, the TKB levels were significantly higher in the ARNI group than in the control group (Fig. 2).

### Clinical factors at three months influencing the %ΔTKB level

To clarify the clinical factors contributing to the changes in TKB levels, we performed a simple regression analysis among the clinical factors at three months later and the %ΔTKB level (Table 2). The LVEF was significantly negatively correlated with the %ΔTKB (*P* = 0.007), and the non-esterified fatty acid (NEFA) levels were significantly positively correlated with the %ΔTKB (*P* < 0.001).

**Table 2:** The results of a simple regression analysis to identify the clinical factors at 3 months influencing the %ΔTKB level (n = 46).

	Standard regression coefficients	Standard error	95% CI	P
Age (years)	0.307	2.660	− 5.054 to 5.667	0.909
BMI (kg/m^2^)	5.677	7.949	− 10.343 to 21.697	0.479
SBP (mmHg)	− 3.885	2.493	− 8.910 to 1.139	0.126
DBP (mmHg)	− 2.082	2.591	− 7.304 to 3.140	0.426
Heart rate (beats per minute)	0.053	2.521	− 5.027 to 5.133	0.983
LVEF (%)	− 7.132	2.530	− 12.237 to − 2.026	0.007
FBS (mg/dL)	2.896	1.616	− 0.360 to 6.153	0.080
Fasting blood insulin (µU/mL)	4.337	9.661	− 15.133 to 23.807	0.656
HbA1c (%)	78.271	52.921	− 28.384 to 184.926	0.146
TG (mg/dL)	0.380	0.617	− 0.863 to 1.623	0.541
HDL (mg/dL)	1.333	2.169	− 3.038 to 5.705	0.542
LDL (mg/dL)	0.062	1.097	− 2.149 to 2.274	0.955
AST (U/L)	0.433	5.161	− 9.996 to 10.863	0.933
ALT (U/L)	− 1.732	3.210	− 8.219 to 4.755	0.592
LDH (U/L)	− 2.220	1.106	− 4.454 to 0.015	0.051
ALP (U/L)	− 0.955	1.442	− 3.868 to 1.959	0.512
γ-GTP (U/L)	0.524	1.056	− 1.610 to 2.658	0.623
TB (mg/dL)	51.981	131.100	− 212.234 to 316.197	0.694
Albumin (g/dL)	34.844	91.171	− 148.899 to 218.587	0.704
eGFR (mL/min/1.73 m^2^)	− 0.112	1.645	− 3.428 to 3.204	0.946
UA (mg/dL)	13.418	22.641	− 32.212 to 59.048	0.556
BNP (pg/mL)	0.143	0.384	− 0.630 to 0.917	0.711
NEFA (μEq/L)	0.463	0.092	0.277 to 0.649	< 0.001

TKB, total ketone body; CI, confidence interval; BMI, body mass index; SBP, systolic blood pressure; DBP, diastolic blood pressure; LVEF, left ventricular ejection fraction; FBS, fasting blood sugar; Hb, hemoglobin; TG, triglyceride; HDL, high-density lipoprotein cholesterol; LDL, low-density lipoprotein cholesterol; AST, aspartate aminotransferase; ALT, alanine aminotransferase; LDH, lactate dehydrogenase; ALP, alkaline phosphatase; γ-GTP, γ-glutamyltranspeptidase; TB, total bilirubin; eGFR, estimated glomerular filtration rate; UA, uric acid; BNP, B-type natriuretic peptide; NEFA, non-esterified fatty acid.

Based on the results of a simple regression analysis, the multiple regression analysis for the %ΔTKB level included the initiation of ARNI, the LVEF, and the NEFA level as independent variables. As shown in Table 3, the multiple regression analysis revealed that the initiation of ARNI and the NEFA level was independently and significantly associated with the %ΔTKB (initiation of ARNI: *P* = 0.017, NEFA level: *P* < 0.001), while the LVEF was not significantly correlated with the %ΔTKB.

**Table 3:** Results of the multiple regression analysis to identify the clinical factors at 3 months influencing %ΔTKB level.

	Coefficient	Standard Error	t value	*P* value	95% CI
Initiation of ARNI	123.913	49.623	2.50	0.017	23.622 to 224.205
LVEF (%)	− 2.455	2.201	− 1.12	0.271	− 6.903 to 1.993
NEFA (μEq/L)	0.397	0.088	4.52	< 0.001	0.219 to 0.574

Adjusted R^2^ = 0.4819.

TKB, total ketone body; CI, confidence interval; ARNI, angiotensin receptor-neprilysin inhibitor; LVEF, left ventricular ejection fraction; NEFA, non-esterified fatty acid.

### The relationship among the %ΔB-type natriuretic peptide (BNP) level, %⊿NEFA level and %⊿TKB level

To clarify the relationship among the %ΔBNP level, %⊿NEFA level and %⊿TKB level, we performed a simple regression analysis of the %ΔBNP with the %ΔNEFA, and of the %ΔNEFA with the %ΔTKB. There was no significant relationship between the %ΔBNP and the %ΔNEFA (*P* = 0.657). However, the %ΔNEFA was significantly related with the %ΔTKB (*P* < 0.0001) (Supplementary Table S11).

### A comparison of blood TKB levels excluding patients who were taking SGLT2 inhibitors

At baseline, there were four patients (two patients in the ARNI group and two patients in the control group) who were taking SGLT2 inhibitors, as shown in Table 1 and Supplementary Table S3. These patients had already been taking SGLT2 inhibitors for more than four weeks prior to baseline. To eliminate the effect of SGLT2 inhibitors, we excluded the four patients who were taking SGLT2 inhibitors, and compared the blood TKB levels in the remaining patients of the group (ARNI group: n = 21, Control group: n = 21). In the control group, there was no marked difference in TKB levels between baseline and 3 months later. In the ARNI group, the TKB levels were significantly higher at 3 months than at baseline (baseline to 3 months: 68 [51, 97] to 92 [67, 225] μmol/L, *P* < 0.01). In addition, at baseline, there was no marked difference in TKB levels between the 2 groups, but after 3 months, the TKB levels were significantly higher in the ARNI group than in the control group (92 [67, 225] vs. 54 [44, 74] μmol/L, *P* < 0.01). These results were the same as those found in the whole analysis, which included patients taking SGLT2 inhibitors.

## Discussion

The current study is the first to evaluate the effect of ARNI administration on the blood ketone body levels, and the results showed that the administration of ARNI increased the TKB levels in pre-heart failure/heart failure patients. In addition, ARNI administration and an increase in the blood non-esterified fatty acid (NEFA) levels at 3 months were factors that increased the percentage changes in the TKB levels from baseline to 3 months (%ΔTKB level).

In this study, we found that ARNI administration caused an increase in TKB levels in pre-heart failure/heart failure patients. In the liver mitochondria, ketone bodies are produced from acetyl coenzyme A (CoA), which is derived from the β-oxidation of free fatty acids^21,22^. Although it was reported that blood TKB levels in patients with heart failure were elevated with the deterioration of hemodynamics^23^, we recently demonstrated that TKB levels closely correlate with NP levels rather than with the severity of their heart failure^24^. Previous reports demonstrated that, increased NP in heart failure patients activates lipolysis in white adipose tissue (WAT)^25–27^. Another report showed that cold exposure increases the A-type natriuretic peptide (ANP) receptor guanylyl cyclase A (GCA) gene and protein expression in WAT, thus rendering mouse adipocytes responsiveness to ANP-mediated lipolysis^22^. In addition, we previously demonstrated that exogenous ANP ameliorates high-fat-diet (HFD)-induced insulin resistance by promoting adipose tissue browning as well as by attenuating hepatic steatosis^16^. It is most likely that the elevation of NPs due to ARNI administration activates lipolysis in adipose tissue, thereby increasing the concentration of fatty acids in the blood, which are taken up by the liver, resulting in ketone body production. In addition, a previous report showed that insulin reduction results in the mobilization of free fatty acids from adipose tissue to the liver^28^. The present study showed that blood insulin levels were significantly reduced between baseline and three months after the initiation of ARNI. Therefore, insulin reduction was also found to contribute to lipolysis in adipose tissue and consequently it therefore promotes ketone body production.

At baseline, insulin resistance in the ARNI group was higher than in the control group. Insulin resistance is closely related to the pathogenesis of heart failure, and insulin resistance itself is a major factor in the worsening of heart failure^29^. Patients with increased insulin resistance, which occurs in patients suffering from obesity and diabetes, show the suppression of NPs secretion and an accelerated NPs degradation, and consequently, unexpectedly demonstrate low NPs levels in proportion to the heart failure severity^30–32^. Therefore, ARNI administration to such "low NPs" patients tends to cause a more effective endogenous NPs elevation, thus resulting in an increase in the blood ketone body concentrations, and consequently thereby demonstrates cardioprotective effects.

In the present study, there was no significant increase in the BNP level with the initiation of ARNI. Furthermore, although the %ΔNEFA was significantly related to the %ΔTKB, there was no significant relationship between the %ΔBNP and the %ΔNEFA, and between %ΔBNP and %ΔTKB. BNP shows a lower affinity for neprilysin than ANP, so ARNI increases the concentration of ANP levels rather than the BNP levels^33^. We lack ANP data in the present study, but we speculate that the initiation of ARNI would significantly increase the ANP level, and there may be significant relationships between %ΔANP and %ΔNEFA and between %ΔANP and %ΔTKB. Furthermore, C-type natriuretic peptide (CNP) and other peptides may also be involved in the elevation of NEFA and TKB, which is an interesting issue to be further investigated in the future.

It is possible that there is another pathway by which ARNI initiation affects TKB levels. In adipocytes, NPs activate peroxisome proliferator-activated receptor γ coactivator (PGC)-1α and uncoupling protein 1 (UCP1) expression, induce mitochondriogenesis, and increase uncoupled and total respiration^15^. The nuclear receptor peroxisome proliferator activated receptor α (PPARα) is an important element in the metabolic network, where it participates in signaling driven by the main nutrient sensors, such as PGC-1α, and induces hormonal mediators, such as fibroblast growth factor 21 (FGF21), which is produced in the liver^34–36^. Furthermore, in human hepatocytes, PPARα is a strong inducer of 3-hydroxymethylglutaryl-CoA synthase 2 (HMGCS2), a rate-limiting enzyme of endogenous ketogenesis^37^. Therefore, ARNI may be involved in the induction of HMGCS2 via the PGC-1α-PPARα-FGF21 axis and increased ketone body production.

Several limitations associated with the present study warrant mention. First, it was a retrospective, single-center study with a small sample size, and not only heart failure patients but also pre-heart failure patients with low BNP levels (e.g. Stage B heart failure) were included in this study population. Second, the final decision to switch from ACE inhibitors or ARBs to ARNI was dependent on the physician’s and patient’s will (e.g. some patients refused for economic reasons), which could have influenced their metabolic status per se. Third, at baseline, the type and internal dose of ACE inhibitors or ARBs varied from case to case, as shown in Table S3, and variations in these medications may also influence some clinical factors.

In conclusion, the administration of ARNI increased the TKB levels in pre-heart failure/heart failure patients. In addition, ARNI administration and an increase in blood NEFA levels at 3 months independently affected the %ΔTKB level. These results indicate that ARNI induces a mild elevation of blood TKB levels in pre-heart failure/heart failure patients, which may result in cardioprotective effects similar to those of SGLT2 inhibitors. These results may also shed light on the potential mechanism of ARNI as a treatment for heart failure via the activation of ketone body metabolism.

## Methods

### Patient population

**Figure 3:**
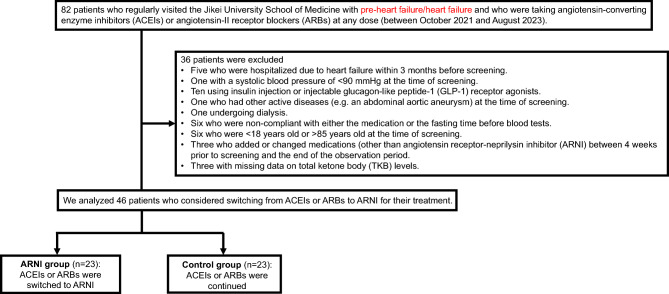
Patient flow chart for the present study. We excluded 36 patients, ultimately analyzing 46 patients who considered switching from an ACE inhibitor or ARB to ARNI. ACE, angiotensin-converting enzyme; ARB, angiotensin receptor blocker; ARNI, angiotensin receptor-neprilysin inhibitor.

This study is a retrospective observational study, and the study population consisted of 82 patients who regularly visited the outpatient department of Jikei University School of Medicine with pre-heart failure/heart failure between October 2021 and August 2023 and were taking ACE inhibitors or ARBs at any dose (Fig. 3). ACE inhibitors or ARBs were initiated in all patients in the present study for at least four weeks before screening. We excluded 36 patients (as described below) and ultimately analyzed 46 patients in the present study.

Patients who switched from ACE inhibitors or ARBs to ARNI were classified into the ARNI group (n = 23), whereas those who continued treatment with ACE inhibitors or ARBs were classified into the control group (n = 23). ARNI treatment was initially started at 100 or 200 mg/day by carefully monitoring the blood pressure and other clinical parameters. For all patients in the present study, no other medications were added or changed from four weeks prior to screening to the end of the observation period, as previously described^20^.

The exclusion criteria were as follows:

i. Patients hospitalized due to heart failure within 3 months before screening. (n = 5)
ii. Patient with a systolic blood pressure of < 90 mmHg at the time of screening (n = 1)
iii. Patients using insulin injection or injectable glucagon-like peptide-1 (GLP-1) receptor agonists (n = 10)
iv. Patient with other active diseases (e.g. an abdominal aortic aneurysm) at the time of screening (n = 1)
v. Patient undergoing dialysis (n = 1)
vi. Patients who were non-compliant with either the medication or the fasting time before blood tests (n = 6)
vii. Patients < 18 years old or > 85 years old at the time of screening (n = 6)
viii. Patients who added or changed medications (other than ARNI) between 4 weeks prior to screening and the end of the observation period (n = 3)
ix. Patients with missing data on TKB levels (n = 3)

This study was conducted in accordance with the principles expressed in the Declaration of Helsinki and was approved by the medical ethics committee of Jikei University School of Medicine [34-046(11191)]. This was a retrospective study, and the need for informed consent from all subjects and/or their legal guardian(s) was waived by the medical ethics committee. Instead of obtaining informed consent from each patient, we posted a notice about the study design and contact information at a public location in our institution, according to our routine ethical regulations. In this public notification, we ensured that patients had the opportunity to refuse to participate (opt-out) in the study, as previously described^20^.

### Data collection

The clinical characteristics of the patients were measured at baseline and three months later and were retrospectively collected from their medical records. The baseline in this study was the point at which the decision to switch from an ACE inhibitor or ARB to an ARNI was considered. At the baseline, in the control group, the patients continued on ACE inhibitors or ARBs without switching to ARNI either based on the physician's decision or the patient's will. The LVEF was measured using transthoracic echocardiography (TTE). Blood pressure was measured at rest in the outpatient department. Blood samples were collected in the morning after at least 11 h of fasting. We defined the percentage change from baseline to 3 months after the TKB level as %ΔTKB (%ΔTKB [%] = 100 × [TKB at 3 months–TKB at baseline]/TKB at baseline). Similarly, we defined %ΔBNP (%ΔBNP [%] = 100 × [BNP at 3 months–BNP at baseline]/BNP at baseline), %ΔNEFA (%ΔNEFA [%] = 100 × [NEFA at 3 months–NEFA at baseline]/NEFA at baseline), and %ΔLVEF (%ΔLVEF [%] = 100 × [LVEF at 3 months–LVEF at baseline]/LVEF at baseline). Blood TKB levels were measured using an enzyme cycling method with a TKB-L test kit (“Kainos” Laboratories, Inc., Tokyo, Japan).

### Statistical analyses

Data are expressed as the mean ± standard error (SE) or as the median (25th, 75th percentile) for significantly skewed variables. For continuous variables, differences between two groups were evaluated using either an unpaired Student’s *t*-test or the Mann–Whitney rank-sum test, and differences within groups were evaluated using a paired *t*-test or Wilcoxon’s signed-rank test, as previously described^20^. For discrete variables, which were expressed as counts and percentages, differences between the two groups were analyzed using the chi-square test, unless the expected value in any cell was < 5, in which case Fisher’s exact test was used. The LVEF measured using TTE were compared among the three time points (baseline, 3 months, and 1 year) using a repeated measure analysis of variance followed by Bonferroni’s test. A multiple linear regression analysis with the forced entry method was performed to determine the contribution of various clinical variables to the %ΔTKB levels. When conducting the multivariate logistic regression analysis, we included predictors with a *P* value < 0.05 in the single regression analysis.

All statistical analyses were performed using the STATA software program (version 16.0; STATA Corp., College Station, TX, USA). Statistical significance was set at *P* < 0.05.

### Supplementary Information

Supplementary Information.

## Data Availability

The data that support the findings of this study are not openly available due to reasons of sensitivity and are available from the corresponding author upon reasonable request.
