# Supplementary material for: Effects of angiotensin receptor-neprilysin inhibitor on ketone body metabolism in pre-heart failure/heart failure patients
Source: Sci Rep. 2024 Jul 17;14:16493. doi: 10.1038/s41598-024-67524-6 (PMC11255280; doi:10.1038/s41598-024-67524-6)

**Supplementary Information**

**Effects of Angiotensin Receptor-Neprilysin Inhibitor on Ketone Body Metabolism in Pre-Heart Failure/Heart Failure Patients**

Yusuke Kashiwagi*^1^, Tomohisa Nagoshi^1^, Yoshiro Tanaka^1^, Yuhei Oi^1^, Haruka Kimura^1^, Kazuo Ogawa^1^, Makoto Kawai ^1^, Michihiro Yoshimura^1^

^1^ Division of Cardiology, Department of Internal Medicine, The Jikei University School of Medicine, Tokyo, Japan

**Supplemental Table S1. NYHA classifications of the patients (n=46) at baseline.**

| NYHA class | **ARNI group (n=23)** | **Control group (n=23)** |
| --- | --- | --- |
| Class Ⅰ (n) | 21 | 22 |
| Class Ⅱ (n) | 2 | 1 |
| Class Ⅲ (n) | 0 | 0 |
| Class Ⅳ(n) | 0 | 0 |

NYHA, New York Heart Association; ARNI, angiotensin receptor-neprilysin inhibitor.

**Supplemental Table S2. ACCF/AHA stages of heart failure of the patients.**

|  | **ARNI group (n=23)** | **Control group (n=23)** |
| --- | --- | --- |
| Stage A (n) | 0 | 0 |
| Stage B (n) | 17 | 16 |
| Stage C (n) | 6 | 7 |
| Stage D (n) | 0 | 0 |

ACCF, American College of Cardiology Foundation; AHA, American Heart Association; ARNI, angiotensin receptor-neprilysin inhibitor.

**Supplemental Table S3. Oral medication of the patients at baseline. (ACE Inhibitor or ARB, and oral antihyperglycemic drug)**

| Case | Group | ACE Inhibitor or ARB | Oral antihyperglycemic drug |
| --- | --- | --- | --- |
| 1 | ARNI | Enalapril 2.5 mg/day | None |
| 2 | ARNI | Telmisartan 40 mg/day | None |
| 3 | ARNI | Alacepril 25 mg/day | None |
| 4 | ARNI | Enalapril 2.5 mg/day | None |
| 5 | ARNI | Losartan 25 mg/day | Sitagliptin 100 mg, Empagliflozin 10 mg |
| 6 | ARNI | Imidapril 5 mg/day | Linagliptin 5 mg |
| 7 | ARNI | Enalapril 2.5 mg/day | None |
| 8 | ARNI | Enalapril 5 mg/day | None |
| 9 | ARNI | Enalapril 5 mg/day | None |
| 10 | ARNI | Enalapril 2.5 mg/day | None |
| 11 | ARNI | Losartan 50 mg/day | None |
| 12 | ARNI | Enalapril 5 mg/day | None |
| 13 | ARNI | Telmisartan 40 mg/day | None |
| 14 | ARNI | Olmesartan 20 mg/day | Metformin 100 mg, Glimepiride 1 mg |
| 15 | ARNI | Olmesartan 20 mg/day | None |
| 16 | ARNI | Irbesartan 50 mg | None |
| 17 | ARNI | Azilsartan 20 mg/day | None |
| 18 | ARNI | Imidapril 2.5 mg/day | Tofogliflozin 20 mg, Metformin 250 mg |
| 19 | ARNI | Enalapril 5 mg/day | None |
| 20 | ARNI | Azilsartan 20 mg/day | None |
| 21 | ARNI | Valsartan 40 mg | None |
| 22 | ARNI | Imidapril 2.5 mg/day | Sitagliptin 50 mg, Voglibose 0.4 mg |
| 23 | ARNI | Azilsartan 20 mg/day | None |
| 24 | Control | Losartan 25 mg/day | None |
| 25 | Control | Valsartan 80 mg | None |
| 26 | Control | Azilsartan 40 mg/day | None |
| 27 | Control | Enalapril 2.5 mg/day | None |
| 28 | Control | Olmesartan 20 mg/day | None |
| 29 | Control | Azilsartan 20 mg/day | None |
| 30 | Control | Enalapril 5 mg/day | None |
| 31 | Control | Losartan 50 mg/day | None |
| 32 | Control | Enalapril 2.5 mg/day | None |
| 33 | Control | Enalapril 1.25 mg/day | Empagliflozin 10 mg, Linagliptin 5 mg |
| 34 | Control | Losartan 25 mg/day | None |
| 35 | Control | Enalapril 5 mg/day | None |
| 36 | Control | Enalapril 5 mg/day | None |
| 37 | Control | Telmisartan 80 mg | None |
| 38 | Control | Enalapril 2.5 mg/day | None |
| 39 | Control | Enalapril 1.25 mg/day | None |
| 40 | Control | Valsartan 20 mg | Metformin 1500 mg, Sitagliptin 50 mg, Dapagliflozin 5 mg |
| 41 | Control | Enalapril 2.5 mg/day | None |
| 42 | Control | Azilsartan 20 mg/day | None |
| 43 | Control | Enalapril 2.5 mg/day | None |
| 44 | Control | Valsartan 40 mg | None |
| 45 | Control | Azilsartan 20 mg/day | None |
| 46 | Control | Azilsartan 40 mg/day | None |

ACE, angiotensin-converting enzyme; ARB, angiotensin receptor blocker; ARNI, angiotensin receptor-neprilysin inhibitor.

**Supplementary Table S4.** **The BNP levels at baseline and 3 months in this study (n=46).**

| **Case** | **Group** | **BNP at baseline (pg/mL)** | **BNP at 3 months (pg/mL)** |
| --- | --- | --- | --- |
| 1 | ARNI | 416.0 | 227.9 |
| 2 | ARNI | 364.6 | 266.8 |
| 3 | ARNI | 91.4 | 164.2 |
| 4 | ARNI | 39.3 | 18.4 |
| 5 | ARNI | 237.2 | 296.3 |
| 6 | ARNI | 21.7 | 33.7 |
| 7 | ARNI | 69.9 | 55.1 |
| 8 | ARNI | 4.4 | 3.9 |
| 9 | ARNI | 11.2 | 13.4 |
| 10 | ARNI | 116.6 | 134.2 |
| 11 | ARNI | 8.7 | 52.2 |
| 12 | ARNI | 4.2 | 12.5 |
| 13 | ARNI | 26.1 | 21.1 |
| 14 | ARNI | 3.9 | 8.9 |
| 15 | ARNI | 16.2 | 19.0 |
| 16 | ARNI | 10.0 | 64.9 |
| 17 | ARNI | 4.7 | 7.0 |
| 18 | ARNI | 3.9 | 3.9 |
| 19 | ARNI | 17.1 | 22.7 |
| 20 | ARNI | 11.6 | 7.6 |
| 21 | ARNI | 30.5 | 30.3 |
| 22 | ARNI | 59.6 | 48.4 |
| 23 | ARNI | 12.6 | 11.0 |
| 24 | Control | 11.1 | 6.6 |
| 25 | Control | 23.8 | 19.7 |
| 26 | Control | 4.1 | 8.5 |
| 27 | Control | 25.7 | 35.8 |
| 28 | Control | 125.8 | 100.3 |
| 29 | Control | 18.7 | 21.1 |
| 30 | Control | 137.3 | 131.1 |
| 31 | Control | 125.3 | 77.9 |
| 32 | Control | 3.9 | 5.5 |
| 33 | Control | 55.6 | 70.4 |
| 34 | Control | 64.2 | 79.4 |
| 35 | Control | 109.0 | 99.5 |
| 36 | Control | 46.0 | 101.3 |
| 37 | Control | 50.7 | 19.1 |
| 38 | Control | 8.3 | 7.8 |
| 39 | Control | 6.4 | 16.8 |
| 40 | Control | 32.8 | 22.1 |
| 41 | Control | 130.7 | 150.2 |
| 42 | Control | 36.9 | 78.8 |
| 43 | Control | 33.0 | 62.3 |
| 44 | Control | 369.7 | 333.6 |
| 45 | Control | 100.0 | 113.7 |
| 46 | Control | 54.3 | 70.4 |

BNP, B-type natriuretic peptide; ARNI, angiotensin receptor-neprilysin inhibitor.

**Supplementary Table S5. The %ΔBNP levels of the ARNI group and the control group.**

| Characteristics | Number (%), mean ± SE or median (25th, 75th percentile) | | P |
| --- | --- | --- | --- |
|  | **ARNI group (n=23)** | **Control group (n=23)** |  |
| %ΔBNP (%) | 15.1 (-19.2, 55.3) | 13.7 (-17.2, 41.0) | 0.921 |

BNP, B-type natriuretic peptide; ARNI, angiotensin receptor-neprilysin inhibitor.

%ΔBNP (%) = 100 × (BNP at 3 months – BNP at baseline) / BNP at baseline.

**Supplementary Table S6.** **The results of a simple regression analysis of the BNP level at 3 months with the TKB level at 3 months (n=46).**

|  | Standard regression coefficients | Standard error | 95% CI | P |
| --- | --- | --- | --- | --- |
| BNP level at 3 months (pg/mL) | -0.317 | 0.677 | -1.682 to 1.048 | 0.642 |

B-type natriuretic peptide; TKB, total ketone body; BNP.

**Supplementary Table S7.** **The results of a simple regression analysis of the BNP level at 3 months with the %ΔTKB (n=46).**

|  | Standard regression coefficients | Standard error | 95% CI | P |
| --- | --- | --- | --- | --- |
| BNP level at 3 months (pg/mL) | 0.143 | 0.384 | -0.630 to 0.917 | 0.711 |

BNP, B-type natriuretic peptide; TKB, total ketone body.

%ΔTKB (%) = 100 × (TKB at 3 months – TKB at baseline) / TKB at baseline.

**Supplementary Table S8.** **The results of a simple regression analysis of the %ΔBNP with the %ΔTKB (n=46).**

|  | Standard regression coefficients | Standard error | 95% CI | P |
| --- | --- | --- | --- | --- |
| %ΔBNP (%) | -0.076 | 0.258 | -0.596 to 0.445 | 0.771 |

BNP, B-type natriuretic peptide; TKB, total ketone body.

%ΔBNP (%) = 100 × (BNP at 3 months – BNP at baseline) / BNP at baseline.

%ΔTKB (%) = 100 × (TKB at 3 months – TKB at baseline) / TKB at baseline.

**Supplementary Table S9. The NEFA level at 3 months and the %ΔNEFA levels of the ARNI group and the control group.**

| Characteristics | Number (%), mean ± SE or median (25th, 75th percentile) | | P |
| --- | --- | --- | --- |
|  | **ARNI group (n=23)** | **Control group (n=23)** |  |
| NEFA at 3 months　(μEq/L) | 478 (401, 797) | 540 (309, 634) | 0.362 |
| %ΔNEFA (%) | 21.1 (-29.0, 40.5) | -13.2 (-24.7, 15.9) | 0.144 |

NEFA, non-esterified fatty acid; ARNI, angiotensin receptor-neprilysin inhibitor.

%ΔNEFA (%) = 100 × (NEFA at 3 months – NEFA at baseline) / NEFA at baseline.

**Supplementary Table S10.** **The results of a simple regression analysis of the %ΔBNP with the %ΔNEFA (n=46).**

|  | Standard regression coefficients | Standard error | 95% CI | P |
| --- | --- | --- | --- | --- |
| %ΔBNP (%) | -0.026 | 0.058 | -0.142 to 0.090 | 0.657 |

BNP, B-type natriuretic peptide; NEFA, non-esterified fatty acid.

%ΔBNP (%) = 100 × (BNP at 3 months – BNP at baseline) / BNP at baseline.

%ΔNEFA (%) = 100 × (NEFA at 3 months – NEFA at baseline) / NEFA at baseline.

**Supplementary Table S11.** **The results of a simple regression analysis of the %ΔNEFA with the %ΔTKB (n=46).**

|  | Standard regression coefficients | Standard error | 95% CI | P |
| --- | --- | --- | --- | --- |
| %ΔNEFA (%) | 2.564 | 0.553 | 1.450 to 3.678 | <0.0001 |

NEFA, non-esterified fatty acid; TKB, total ketone body.

%ΔNEFA (%) = 100 × (NEFA at 3 months – NEFA at baseline) / NEFA at baseline.

%ΔTKB (%) = 100 × (TKB at 3 months – TKB at baseline) / TKB at baseline.

**Supplementary Table S12. The ARNI dose (initial and final) and TKB levels at 3 months of the ARNI group.**

| **Case** | **Initial dose (mg/day)** | **Final dose (at 3 months) (mg/day)** | **TKB level (μmol/L) at 3 months** |
| --- | --- | --- | --- |
| 1 | 100 | 200 | 270 |
| 2 | 100 | 100 | 375 |
| 3 | 100 | 100 | 165 |
| 4 | 100 | 100 | 78 |
| 5 | 100 | 100 | 46 |
| 6 | 100 | 100 | 1778 |
| 7 | 100 | 100 | 1111 |
| 8 | 100 | 100 | 177 |
| 9 | 100 | 100 | 57 |
| 10 | 100 | 100 | 196 |
| 11 | 100 | 100 | 59 |
| 12 | 100 | 100 | 102 |
| 13 | 200 | 200 | 225 |
| 14 | 200 | 400 | 57 |
| 15 | 100 | 200 | 34 |
| 16 | 100 | 100 | 92 |
| 17 | 200 | 200 | 604 |
| 18 | 100 | 100 | 1316 |
| 19 | 100 | 100 | 88 |
| 20 | 200 | 200 | 75 |
| 21 | 100 | 100 | 85 |
| 22 | 200 | 200 | 61 |
| 23 | 200 | 200 | 67 |

ARNI, angiotensin receptor-neprilysin inhibitor; TKB, total ketone body.

**Supplementary Table S13. The results of a simple regression analysis of the ARNI dose (initial and final) with the TKB level at 3 months (n=23).**

|  | Standard regression coefficients | Standard error | 95% CI | P |
| --- | --- | --- | --- | --- |
| ARNI dose (initial) | -1.099 | 1.204 | -3.602 to 1.404 | 0.372 |
| ARNI dose (final) | -0.921 | 0.730 | -2.439 to 0.597 | 0.221 |

ARNI, angiotensin receptor-neprilysin inhibitor; TKB, total ketone body.

**Supplemental Table S14. Classification of patients with heart failure (including pre-heart failure) according to LVEF (n=46).**

| **Based on LVEF (n)** | **ARNI group (n=23)** | **Control group (n=23)** |
| --- | --- | --- |
| HFpEF (≥50%) (n) | 13 | 21 |
| HFmrEF (40%-49%) (n) | 6 | 1 |
| HFrEF (<40%) (n) | 4 | 1 |

LVEF, left ventricular ejection fraction; ARNI, angiotensin receptor-neprilysin inhibitor; HFpEF, heart failure with preserved ejection fraction; HFmrEF, heart failure with mid-range ejection fraction; HFrEF, heart failure with reduced ejection fraction.

**Supplementary Table S15.** **The results of a simple regression analysis of the %ΔLVEF with the %ΔTKB (n=46).**

|  | Standard regression coefficients | Standard error | 95% CI | P |
| --- | --- | --- | --- | --- |
| %ΔLVEF (%) | -1.842 | 1.277 | -4.416 to 0.732 | 0.156 |

LVEF, left ventricular ejection fraction; TKB, total ketone body.

%ΔLVEF (%) = 100 × (LVEF at 3 months – LVEF at baseline) / LVEF at baseline.

%ΔTKB (%) = 100 × (TKB at 3 months – TKB at baseline) / TKB at baseline.

**Supplementary Figure. Comparison of LVEF among baseline, 3 months, and 1 year follow-up.** (a) ARNI group (n=15), (b) Control group (n=12). LVEF, left ventricular ejection fraction; ARNI, angiotensin receptor-neprilysin inhibitor; N.S., not significant.


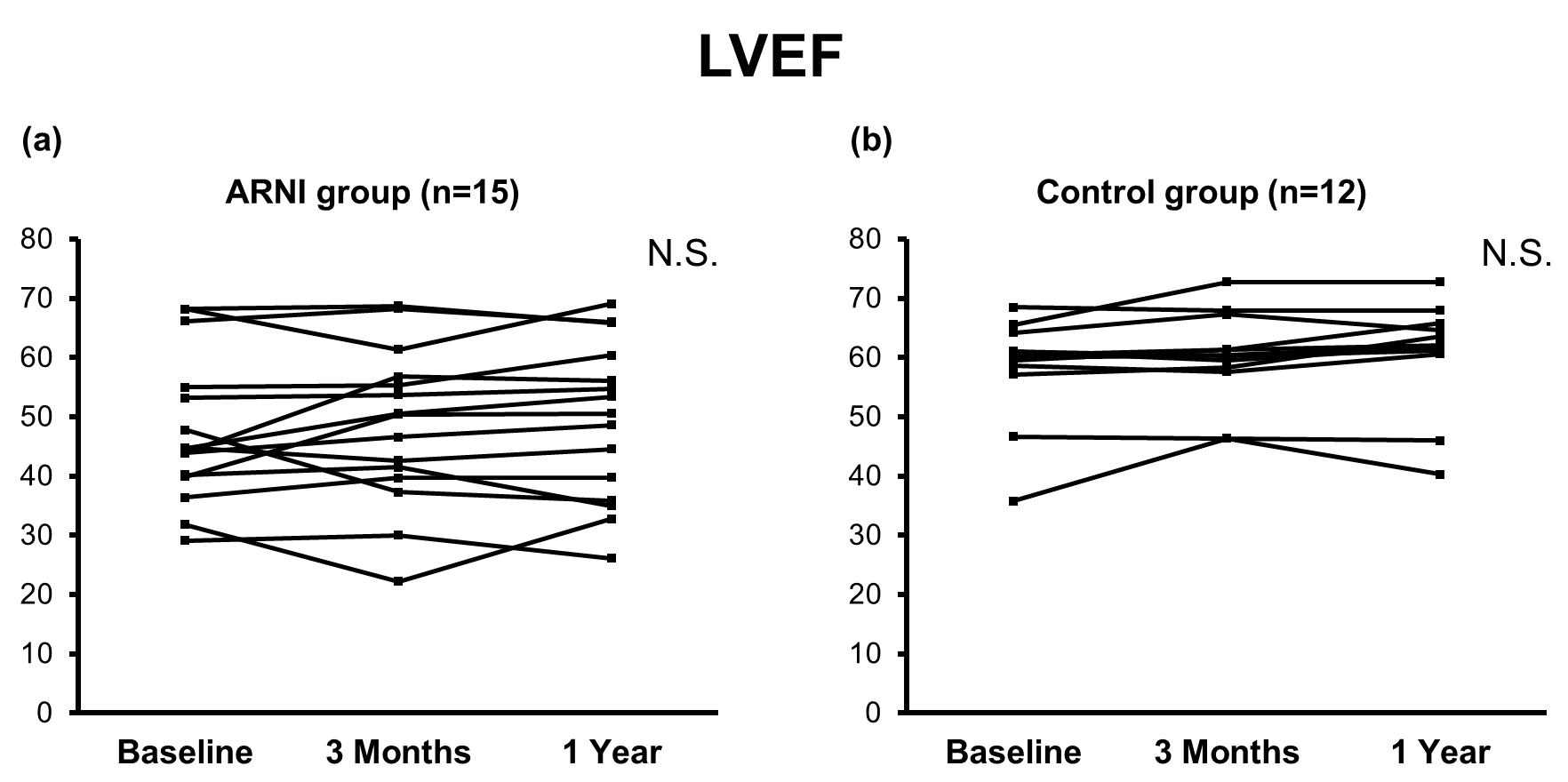

Supplement: Supplementary file 1 — Supplementary Information. [file 41598_2024_67524_MOESM1_ESM.docx]
